# Changes in Plasma Concentration of Free Proteinogenic and Non-Proteinogenic Amino Acids in High-Performance Sprinters over a 6-Month Training Cycle

**DOI:** 10.3390/jcm13175300

**Published:** 2024-09-06

**Authors:** Krzysztof Kusy, Jan Matysiak, Ewa Anna Zarębska, Agnieszka Klupczyńska-Gabryszak, Monika Ciekot-Sołtysiak, Szymon Plewa, Zenon J. Kokot, Paweł Dereziński, Jacek Zieliński

**Affiliations:** 1 Department of Athletics, Strength and Conditioning, Poznan University of Physical Education, Królowej Jadwigi Street 27/39, 61-871 Poznań, Poland; zarebska@awf.poznan.pl (E.A.Z.); ciekot@awf.poznan.pl (M.C.-S.); jzielinski@awf.poznan.pl (J.Z.); 2 Department of Inorganic and Analytical Chemistry, Poznan University of Medical Sciences, ul. Rokietnicka, 60-806 Poznań, Poland; jmatysiak@ump.edu.pl (J.M.); aklupczynska@ump.edu.pl (A.K.-G.); splewa@ump.edu.pl (S.P.); z.kokot@akademiakaliska.edu.pl (Z.J.K.); p.derezinski@gmail.com (P.D.); 3 Faculty of Health Sciences, Calisia University, ul. Nowy Świat, 4, 62-800 Kalisz, Poland

**Keywords:** plasma amino acids, speed-power athletes, training, LC-ESI-MS/MS method

## Abstract

**Background/Objectives:** Free amino acids substantially contribute to energy metabolism. Also, their profile may identify (over)training status and effectiveness. The long-term effects of speed-power training on plasma free amino acid (PFAA) profiles are not known. We aimed to observe variations in PFAA levels in high-performance sprinters in a six-month training cycle. **Methods:** Ten male athletes (24.6 ± 3.3 years) were examined during four training phases: transition (1 month), general preparation (2 months), specific preparation (1 month), and pre-competition/competition (2 months). Venous blood was collected at rest, after exhaustive exercise, and recovery. Forty-two PFAAs were analyzed by the LC-ESI-MS/MS method. **Results:** Significant decreases in resting concentrations were observed between the transition and competition phases for glutamine (762 ± 117 vs. 623 ± 53 μmol∙L^−1^; *p* < 0.001, η^2^ = 0.47) and histidine (89 ± 15 vs. 75 ± 10 μmol∙L^−1^; *p* = 0.010, η^2^ = 0.27), whereas β-alanine (30 ± 7 vs. 41 ± 9 μmol∙L^−1^; *p* = 0.024, η^2^ = 016) and sarcosine (3.6 ± 0.4 vs. 4.8 ± 0.6 μmol∙L^−1^; *p* = 0.006, η^2^ = 0.188) levels increased. Between the specific and competition phases, significant decreases in the resting levels of 1-methylhistidine (22.1 ± 19.4 vs. 9.6 ± 8.8 μmol∙L^−1^; *p* = 0.14, η^2^ = 0.19), 3-methylhistidine (7.1 ± 1.5 vs. 6.5 ± 1.6 μmol∙L^−1^; *p* = 0.009, η^2^ = 0.18), citrulline (40 ± 10 vs. 29 ± 4 μmol∙L^−1^; *p* = 0.05, η^2^ = 0.29), and ornithine (74 ± 15 vs. 56 ± 10 μmol∙L^−1^; *p* = 0.015, η^2^ = 185) were noticed. Also, for β-alanine and sarcosine, the pattern of response to exercise strongly changed between the training phases. Blood ammonia levels at exhaustion decreased between the transition and competition phases (32 ± 4 vs. 23 ± 5 μmol∙L^−1^; *p* < 0.001, η^2^ = 0.67), while lactate, the phenylalanine–tyrosine ratio, the glutamine–glutamate ratio, hematological parameters, and cardiorespiratory indices remained at similar levels. **Conclusions:** Speed-power training seems to affect PFAAs involved in skeletal muscle metabolic pathways responsible for neutralizing toxic ammonia (glutamine, arginine, citrulline, ornithine), attenuating the deleterious effects of H^+^ ions (histidine, β-alanine), and reducing exercise-induced protein breakdown (1- and 3-methylhistidine). Our findings suggest that sprint-oriented training supports metabolic pathways that are responsible for the removal of harmful metabolites produced during exercise.

## 1. Introduction

The role of free amino acids during exercise is crucial, despite the fact that their contribution to the pool of total amino acids in the human body is only ~1% [1]. While they do not directly provide energy for active skeletal muscles, they substantially contribute to energy metabolism. They deliver carbon skeletons for tricarboxylic acid (TCA) and ensure the smooth functioning of other metabolic pathways activated during exercise, such as the glucose–alanine cycle, glutamate–glutamine cycle, uric acid cycle, and aspartate–malate shuttle, and are therefore rightly termed as the ‘metabolic currency’ [2,3,4,5,6,7]. Consequently, in a healthy individual, especially a competitive athlete, free amino acids perform key functions during exercise, such as maintaining high concentrations of TCA cycle intermediates, neutralizing toxic ammonia, promoting adenosine triphosphate resynthesis, participating in H^+^ ion buffering, or contributing to the mitochondrial respiratory chain [7,8,9]. Overall, from the perspective of sports practice, specific changes in the free amino acid profile may be indicative of adaptive changes and the effectiveness of structured training programs [10]. Some studies also suggest that the blood free amino acid levels and their ratios may be markers of overtraining in professional athletes, allowing for the detection of amino acid deficiencies and possible health disorders before acute symptoms occur [11,12,13,14,15,16].

The levels of individual free amino acids at rest and in response to exercise are affected by long-term training. It was shown that sports or military training periods of 5–10 months cause significant fluctuations in the amino acid profile, reflecting specific metabolic adaptations [14,17,18,19]. However, the available studies mainly address endurance training, directed at increasing aerobic capacity or overall fitness. There is a lack of research on speed-power athletes who are pursuing different, essentially opposite, training effects aimed at developing anaerobic power, speed, and speed endurance. Thus, their training adaptations can be expected to be associated with a distinctive free amino acid profile. As far as non-endurance athletes are concerned, only Kingsbury et al. [13] and Pitkänen et al. [10] attempted to identify the relationship between speed-power training and blood amino acid levels and found specific changes in the amino acid profile. However, further research is needed to obtain a more complete picture of the specific changes in amino acid profiles in speed-power athletes. Notably, Kingsbury et al. [13] only performed resting measurements during an intense pre-Olympic period in a combined group of male and female athletes (also overtrained ones), including a variety of sports, thus not exclusively speed-power athletes. Pitkänen et al. [10] investigated sprinters and jumpers before and after a short 5-week period of specialized training, including responses to two types of sessions. Thus, neither of these two studies analyzed changes in blood amino acid concentrations over longer (multi-month) periods at critical points in a training cycle, and the response to exercise was not assessed or assessed using a non-standardized exercise. In addition, only a few non-proteinogenic amino acids were included. When analyzing athletes, it is important to also consider a wider set of non-proteinogenic amino acids that have not yet been extensively explored in this cohort. They play a variety of roles during exercise, such as exerting an ergogenic effect, assisting in the removal of toxic ammonia, buffering hydrogen ions, entering the creatine cycle and the mitochondrial respiratory chain, activating the beta-oxidation pathway of hepatic fatty acids, reducing the production of reactive oxygen species, or serving as myokines secreted by skeletal muscle, yet they are not encoded in genes or built into proteins during the translation process [20]. In addition, studies in competitive athletes can provide data to compare amino acid concentrations with other healthy or diseased cohorts.

The aim of this study is to evaluate changes in the plasma profile of a complete set of proteinogenic and a broader range of non-proteinogenic amino acids in speed-power athletes during a six-month training cycle structured according to the principles of periodization in competitive sports [21]. We hypothesize that there will be significant fluctuations in plasma free amino acid (PFAA) concentrations at rest and in response to exercise in speed-power athletes during the main training phases of a half-year cycle.

## 2. Materials and Methods

### 2.1. Participants

Ten highly trained male sprinters, aged 24.6 ± 3.3 years, height 185 ± 5 cm, participated in the study. They competed at the national and international levels in relays of distances 100 m, 200 m, and 4 × 100 m relay. During the study period, their best performance in the indoor 60 m sprint was between 6.63 s and 6.81 s. They were recruited from professional sports clubs and national teams and had been practicing speed and power sports for at least five years (10.2 ± 3.5 years). They underwent regular medical examinations in line with the recommendations of the national athletics federation. Their health throughout the study period was good; moreover, they did not suffer injuries. The athletes in the study never had a positive anti-doping test. Study procedures adhered to the ethical standards of the 1964 Declaration of Helsinki and its subsequent amendments. The study was approved by the Bioethics Committee of the Poznan University of Medical Sciences (decision no. 1252/18 of 6 December 2018). The purpose, risks, and benefits of the study were explained to the athletes, who signed written informed consent before the start of the study. All measurements were conducted in the Human Movement Laboratory ‘LaBthletics’ at the Poznan University of Physical Education, Poznan, Poland. The PFAA assay was performed in the Department of Inorganic and Analytical Chemistry at the Poznan University of Medical Sciences, Poznan, Poland. The study was registered in the Clinical Trial Registry, number NCT05672758, released on 5 January 2023.

### 2.2. Study Design

**Figure 1:**
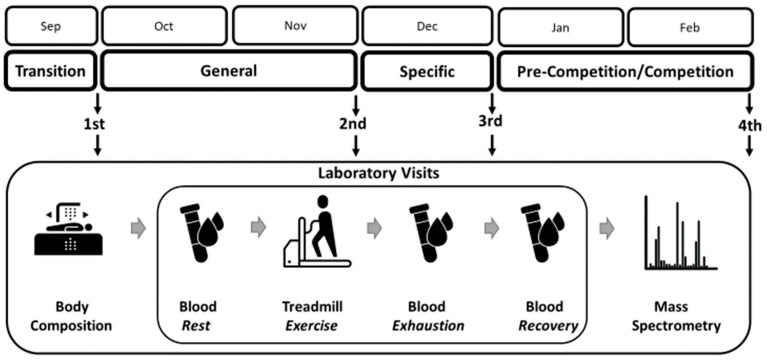
Study design. Athletes were examined after each main training phase of a 6-month cycle. During each single laboratory visit, they underwent body composition measurements and then blood tests at rest, at exhaustion, and during post-exercise recovery. Finally, plasma amino acid concentrations were determined using the liquid chromatography electrospray ionization tandem mass spectrometry technique. Terms of use: The icons used in this figure are licensed under a CC BY 3.0 license. They are attributed to Luis Prado (body composition), Lars Meiertoberens (test tube/blood), Rafiico Creative Studio (treadmill), and Fredrik Edfors (mass spectrometry). The original versions can be found at https://thenounproject.com (accessed on 15 August 2024).

The study design was aligned with the six-month training cycle in preparation for competition during the indoor season. Data were collected on four occasions, which were set after each of the major training phases: (i) a 1-month transition (detraining) phase, (ii) a 2-month general preparation phase, (iii) a 1-month specific preparation phase, and (iv) a 2-month pre-competition and competition indoor season. Each time, the athletes underwent a body composition assessment and performed an incremental exercise test on a mechanical treadmill until exhaustion, with blood draws to determine PFAA levels. The flow of the main research procedures is shown in Figure 1.

### 2.3. Training Description

The training period spanned a six-month cycle, during which the coaches controlled the training loads according to the classic principles of volume and intensity adjustment in the main training phases, sub-phases, macro-, and micro-cycles [21]. In brief, the transition phase consisted in the cessation of training (detraining), with the goal being physical recovery. The general preparation, aimed at improving basic strength and conditioning, consisted mainly of moderate-intensity aerobic running and resistance exercises during the first month. Subsequently, the volume and intensity of training loads were increased. During the specific preparation phase, the training volume was reduced. The focus was on developing speed endurance, speed, and running technique at high intensities. During the pre-competition and competition phases, the total training load was significantly reduced. The emphasis was on highly specific high-intensity and low-volume exercises to achieve and maintain peak performance. 

### 2.4. Dietary Macronutrients

During the pre-competition/competition phase, data on customary diet were collected by recording the foodstuffs, dishes, beverages, and dietary supplements (including protein and amino acids) consumed during a typical week covering weekdays and the weekend. First, participants estimated the quantity of food and drink they consumed at each meal using household measures or grams and verified them with a photo album of food and dishes [22]. A trained sports dietitian then analyzed and calculated energy and macronutrient intake using Dietetyk-2 software, version 2006 (JuMaR, Poznań, Poland). Complete nutritional data suitable for further analysis were obtained from 6 of 10 athletes.

During the entire study period, the athletes continued their usual eating habits. They were required to refrain from consuming excessive fluids and avoid dehydration the day before the test and not to ingest any dietary supplements or aids potentially affecting exercise capacity and blood PFAA concentration. On the test day, athletes arrived at the lab at 7 a.m. after an all-night fast (~12 h). Additionally, before starting procedures, their 48 h dietary intake was examined to detect any abnormal eating patterns that could confound the laboratory assessment (in such a case, the laboratory visit could have been rescheduled).

### 2.5. Anthropometry and Body Composition

Prior to body composition measurement, the athletes urinated and had a bowel movement. A SECA 285 stadiometer (SECA GmbH, Hamburg, Germany) was utilized to measure height and weight. Fat mass, lean body mass, and related indicators were measured using the dual X-ray absorptiometry method (Lunar Prodigy device, enCORE software version 17.50.037; GE Healthcare, Chicago, IL, USA), according to the procedures described previously [23]. The prediction equation by Kim et al. [24] was used to calculate skeletal muscle mass (SMM).

### 2.6. Exercise Test Protocol 

The athletes refrained from performing very strenuous training sessions of high intensity or long duration 24−48 h before the laboratory visit. The tests were conducted between 8 and 11 a.m. The exercise protocol details were previously described by Trinschek et al. [23]. Briefly, an incremental exercise test was conducted (h/p/cosmos^®^ Pulsar treadmill; Sports & Medical GmbH, Nussdorf-Traunstein, Germany), with continuous breath-by-breath gas exchange measurements using a MetaLyzer 3B ergospirometer (Cortex Biophysik GmbH, Leipzig, Germany). The raw data were processed with the MetaSoft Studio 5.1.0 software package (Cortex Biophysik GmbH, Leipzig, Germany). Before each laboratory session and test, the system was calibrated according to the manufacturer’s instructions. The heart rate was monitored using the Polar Bluetooth Smart H6 monitor (Polar Electro Oy, Kempele, Finland). The variable used in this study was maximum oxygen uptake (VO_2_max). Before starting the test, the athlete stood still on the treadmill for 3 min to check the proper operation of the measurement system. After this, the speed increased to 4 km⋅h^−1^ and after another 3 min to 8 km⋅h^−1^. During the subsequent test stages, the speed was increased by 2 km⋅h^−1^ every 3 min until volitional exhaustion. At least three of the following objective criteria had to be met for an athlete to be considered exhausted and to have reached VO_2_max at the end of the test: (i) lack of further increase in VO_2_ despite continued increase in running speed and minute ventilation, (ii) blood lactate concentration ≥ 9 mmol∙L^−1^, (iii) respiratory exchange ratio ≥1.10, and (iv) heart rate equal to ~95% of the actual maximum heart rate (known from previous tests of the same type). The total exercise duration was approximately 20 min. During post-exercise recovery, athletes walked for 3 min at a speed of 4 km⋅h^−1^, and then rested for 30 min in a seated position. The temperature in the laboratory was kept constant at 20–21 °C.

### 2.7. Blood Drawing and Sampling

Blood samples of 2.5 mL each were collected in plasma separation tubes containing EDTA at rest, at the time of volitional exhaustion, and at the 15th and 30th min of post-exercise recovery. For this purpose, a peripheral venous catheter was placed in the antecubital vein. The tubes were centrifuged at 13,000 rpm for 3 min at 4 °C (Universal device, Hettich GmbH, Tuttlingen, Germany). Plasma was then pipetted into 0.5 mL vials and immediately frozen in liquid nitrogen. The V-Vials were stored in an HEF^®^ U410 (New Brunswick Scientific Co., Inc., Edison, NJ, USA) low-temperature freezer at −80 °C until further analysis.

### 2.8. Determination of PFAA Concentration

Forty-two amino acids were assayed, including 20 proteinogenic and 22 non-proteinogenic amino acids. In addition, phenylalanine–tyrosine and glutamine–glutamate ratios were determined to assess the catabolic state caused by possible injury or infection [11,13] and identify potential overreaching [12,15], respectively. A liquid chromatography–electrospray ionization–tandem mass spectrometry (LC-ESI-MS/MS) technique was used: a 1260 Infinity liquid chromatography instrument (Agilent Technologies, Santa Clara, CA, USA) coupled to a 4000 QTRAP mass spectrometer (Sciex, Framingham, MA, USA). The reagent used was aTRAQ^TM^ (Sciex, Framingham, MA, USA). This procedure is characterized by a high degree of specificity, accuracy, and precision in the quantification of PFAA concentrations [25]. The details of the method we used are described in our earlier work [19]. The complete list of PFAAs assayed and their lower limits of quantitation are provided in Appendix A. All PFAA concentrations were corrected for changes in resting plasma volume [26].

### 2.9. Additional Blood Metabolites

Blood lactate was measured using a Biosen C-line (EKF-Diagnostics GmbG, Barleben, Germany). Ammonia concentrations were determined using a PocketChem BA device (Arkray Inc., Kyoto, Japan). Both lactate and ammonia were measured immediately after each blood draw. The creatine kinase level was measured at rest using a Reflotron Plus device (Roche Diagnostics International AG, Basel, Switzerland). Also, a standard blood count, including white and red blood cells, hemoglobin, and hematocrit, was performed using a Sysmex XS-1000i device (Sysmex Europe, Hamburg, Germany). 

### 2.10. Statistical Analysis

The Shapiro–Wilk test showed a normal data distribution for the main variables analyzed; thus, parametric statistical tests were used. A two-way repeated-measures (within–between interaction) analysis of variance (ANOVA) was used to assess the effects of the training phase, test stage, and their interaction on PFAA concentrations. A minimum of 10 participants was set using G*Power software version 3.1.9.6, assuming measurements in 4 consecutive training phases and 4 sampling points during the exercise test, a *p*-value of 0.05, a statistical power of 0.8, and a partial η^2^ of 0.14 [27]. For each variable analyzed by ANOVA, the equality of variance and sphericity were assessed using Levene’s and Mauchly’s tests, respectively. If sphericity was violated, the Greenhouse–Geisser correction was used. To assess the main ANOVA effects and interactions, partial η^2^ was used and considered small (≥0.01), medium (≥0.06), or large (≥0.14). When main effects or interactions showed significance, the Bonferroni correction was applied as a post hoc test. All analyses were considered significant at a *p*-value of less than 0.05. The values of all variables are presented as means and standard deviations.

## 3. Results

### 3.1. Group Description

Weight, body mass index, resting hematological indices, VO_2_max, and HRmax did not change significantly between the training phases. Significant changes in body composition were observed, i.e., fat mass showed a significant decreasing trend, while lean body mass and SMM indicators showed an increasing trend between the transition phase and the competition phase (Table 1).

In the pre-competition/competition period, the average energy intake from food and supplements was 4375 ± 823 kcal·day^−1^ (52.0 ± 7.5 kcal·day^−1^ per kg body weight) in six of ten athletes studied. The estimated daily intake of macronutrients (protein, fat, and carbohydrate) and their contribution to the energy provided are shown in Table 2.

### 3.2. Proteinogenic PFAAs

**Figure 2:**
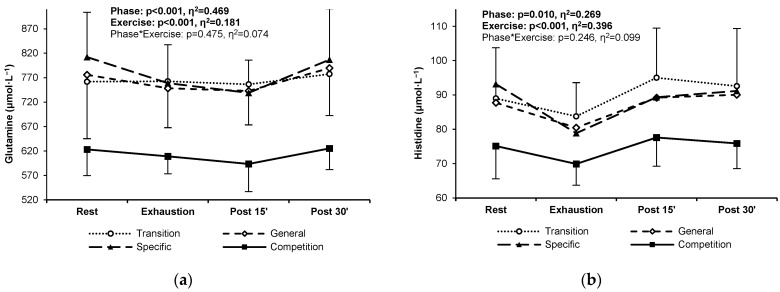
Glutamine (**a**) and histidine (**b**) plasma concentrations in highly trained sprinters at rest, at exhaustion, and during post-exercise recovery in the four main training phases of a 6-month cycle: transition (white circles, dotted line), general preparation (white diamonds, short-dashed line), specific preparation (black triangles, long-dashed line), and competition (black squares, solid line). Significant ANOVA effects of the training phase, exercise/recovery stage, and their interaction are shown in bold.

The resting, exercise, and post-exercise concentrations of two proteinogenic PFAAs, glutamine and histidine, decreased significantly during the competition phase compared to the other training phases (Figure 2a,b). The concentrations of other proteinogenic amino acids did not change significantly over the study period (Appendix B).

### 3.3. Non-Proteinogenic PFAAs

**Figure 3:**
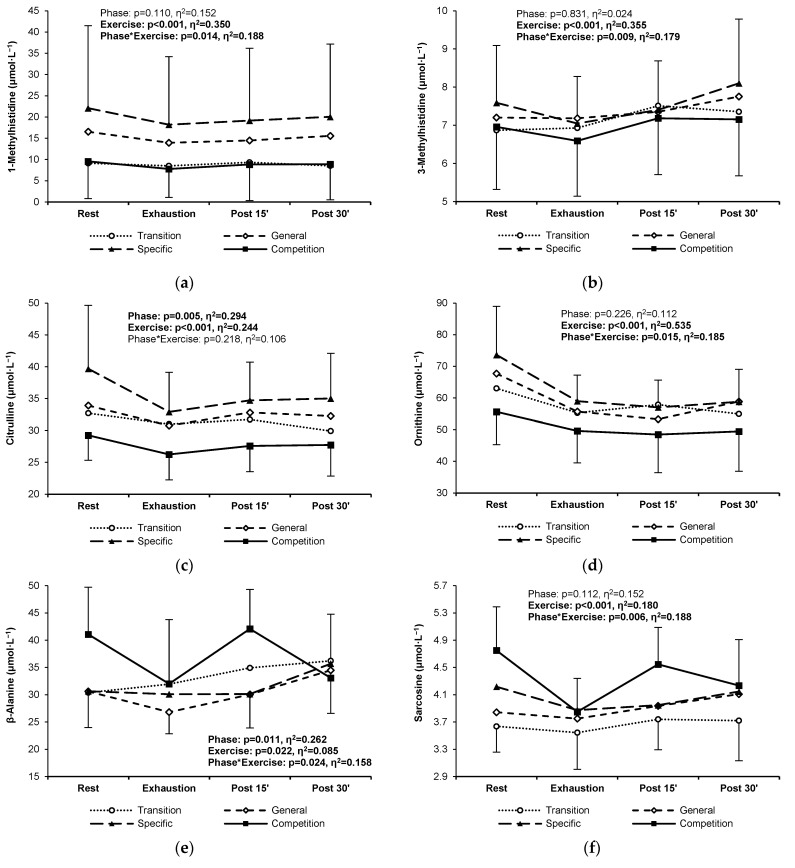
1-Methylhistidine (**a**), 3-methylhistidine (**b**), citrulline (**c**), ornithine (**d**), β-alanine (**e**), and sarcosine (**f**) plasma concentrations in highly trained sprinters at rest, at exhaustion, and during post-exercise recovery in the four main training phases of a 6-month cycle: transition (white circles, dotted line), general preparation (white diamonds, short-dashed line), specific preparation (black triangles, long-dashed line), and competition (black squares, solid line). Significant ANOVA effects of the training phase, exercise/recovery stage, and their interaction are shown in bold.

**Figure 4:**
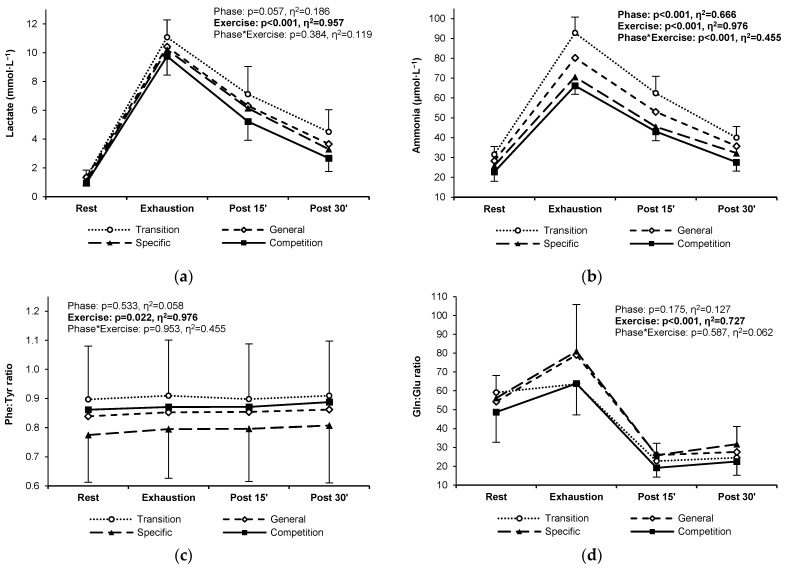
Lactate (**a**), ammonia (**b**), the phenylalanine–tyrosine ratio (**c**), and the glutamine–glutamate ratio (**d**) in highly trained sprinters at rest, at exhaustion, and during post-exercise recovery in the four main training phases of a 6-month cycle: transition (white circles, dotted line), general preparation (white diamonds, short-dashed line), specific preparation (black triangles, long-dashed line), and competition (black squares, solid line). Significant ANOVA effects of the training phase, exercise/recovery stage, and their interaction are shown in bold.

Nine non-proteinogenic PFAAs did not exceed the lower limit of quantitation. These were argininosuccinic acid, cystathionine, anserine, carnosine, homocitrulline, homocystine, phosphoserine, γ-aminobutyric acid, and δ-hydroxylysine. Six non-proteinogenic PFAAs showed a statistically significant change between the four training phases. Levels of 1-methylhistidine were significantly lower in both the transition and competition phases compared to the general and specific phases (Figure 3a). Although average 3-methylhistidine concentrations were similar throughout the training cycle, the pattern of the exercise-induced response was significantly different, i.e., during the post-exercise recovery, a consistent increasing trend was observed in the general and specific phases, whereas the post-exercise levels stabilized in the transition and competition phases (Figure 3b). Citrulline and ornithine levels were significantly lower in the competition phase and higher in the specific preparation phase (Figure 4c,d). The pattern of change in β-alanine and sarcosine concentrations was significantly different between the competition phase and the other phases; i.e., higher resting levels, a more rapid decrease during exercise, and a steeper increase after 15 min of recovery were found in the competition phase (Figure 3e,f). For the other non-protein PFAAs, no significant changes were found between training phases (Appendix B).

### 3.4. Training Status Indicators

The blood lactate response to exercise did not change significantly between the training phases, even if slightly lower post-exercise levels could be noticed in the competition phase (Figure 4a). Blood ammonia levels decreased gradually and significantly between the transition and competition phases (Figure 4b). Resting, exercise, and post-exercise phenylalanine–tyrosine and glutamine–glutamate ratios remained at similar levels throughout the analyzed training cycle (Figure 4c,d). Detailed means and standard deviations for all PFAAs, lactate, and ammonia in the four training phases are provided in Appendix C.

## 4. Discussion

**Figure 5:**
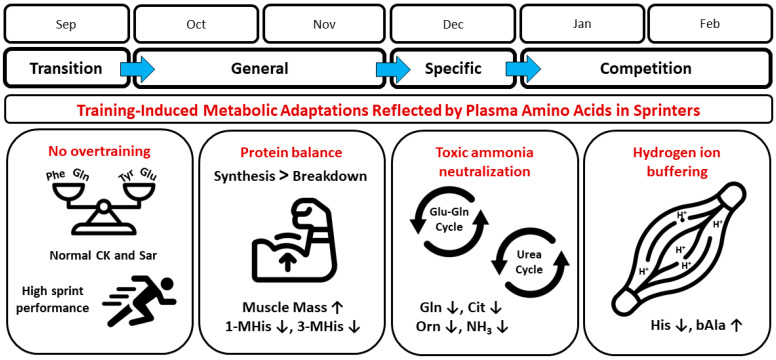
Main metabolic adaptations induced by a 6-month training cycle in sprinters as reflected by plasma free amino acid concentration. Related physiological mechanisms are schematized. See further sections below for explanations. Abbreviations: 1-MHis, 1-methylhistidine; 3-MHis, 3-methylhistidine; bAla, β-alanine; Cit, citrulline; CK, creatine kinase; Gln, glutamine; Glu, glutamate; His, histidine; NH_3_, ammonia; Orn, ornithine; Phe, phenylalanine; Sar, sarcosine; Tyr, tyrosine; ↓, decrease; ↑, increase. Terms of use: The icons used in this figure are licensed under a CC BY 3.0 license. They are attributed to IYIKON (scale), Diego Naive (sprinter), Marz Gallery (biceps), Hrbon (cycles), and Supanut Piyakanont (muscle). The original versions can be found at https://thenounproject.com (accessed on 15 August 2024).

The main findings of this study are as follows: (i) there are significant changes in individual PFAA levels and patterns of response to exercise in speed-power athletes over a six-month structured training cycle, (ii) the changes are most evident in the competition phase, and (iii) training affects both proteinogenic and non-proteinogenic PFAA levels. The most pronounced and statistically significant changes between the four training phases were shown for glutamine, histidine, 1-methylhistidine, 3-methylhistidine, citrulline, ornithine, β-alanine, and sarcosine. To better understand the observed changes in PFAA levels, it is important to consider that training loads in successive phases did not change in a uniformly ‘linear’ manner. In practice, the loads were adjusted according to the general rules used in competitive sports [21]. In broad terms, the changes in the main components of training loads, i.e., exercise volume and intensity, were as follows. During the transition phase, the volume and intensity of exercise were reduced to a minimum (lack of training); thus, the first measurement was a ‘baseline’. In the general preparation phase, both volume and intensity were gradually increased to achieve initial adaptation (second measurement). The special preparation phase was characterized by a gradual reduction in the volume of loads with intensity still increasing (third measurement). In the pre-competition and competition phase, the volume of training was further reduced, accompanied by a very high intensity of loads (limited mainly to specialized exercises), with a relatively low total load, which was conducive to maintaining speed abilities at the highest level (fourth measurement). The main trends in training adaptations between training phases, as reflected by PFAAs, and potentially related physiological mechanisms are summarized in Figure 5 and discussed further below. 

In the only comparable study on speed-power athletes, Pitkänen et al. [10] found a significant tendency for 14 serum amino acid levels to decrease after a 5-week training period in competitive sprinters and jumpers. Specifically, in both our study and theirs, the resting levels of glutamine, histidine, ornithine, and citrulline decreased significantly. They also observed reductions in the resting and post-exercise concentrations of other amino acids. Some differences between the results of our study and those of Pitkänen et al. [10] may be due to several reasons. They did not specify the training phase in the context of the annual training cycle. The sprint performance levels of their athletes appeared to be lower. Furthermore, they did not adjust the amino acid concentrations for changes in plasma volume, as in our study, determined serum amino acid concentrations, used different analytical methods (serum and ion exchange chromatography), and finally used entire training sessions as a ‘test exercise’. However, despite these purely technical differences, there is a basic convergence between the two studies; i.e., they show that specialized speed-power training leads to a reduction in the concentration of individual free amino acids circulating in the blood.

Very short training periods, from a few days to two weeks, may not be sufficient to cause significant changes in plasma PFAAs [16,28], whereas multi-month training clearly affects the PFAA profile [12,19]. However, the nature and strength of the training stimuli, the physiological state of the athlete between the phases (overlap of load effects), and the role and metabolic response of the specific amino acid are other critical factors driving the changes. The individual training phases in our study lasted 1–2 months and the results we obtained indicate that these were sufficient intervals to observe significant trends in high-performance sprinters, in whom the training loads were strong and frequently repeated. Most importantly, the 6-week cycle was a period that included the essential phases of the classic training cycle (mentioned above), thus covering the entire applied variation of training loads.

It also seems that the set of amino acids that undergo significant changes in plasma concentration during a long-term training cycle in athletes may depend on the sport specificity. In an earlier study on endurance athletes tested during a multi-month cycle, significant changes involved a greater number of amino acids, primarily those associated with the TCA cycle and oxidative phosphorylation, i.e., metabolic mechanisms crucial to the development of aerobic capacity [19]. At the same time, as in the sprinters studied here, there were adaptive changes in amino acid levels associated with ammonia neutralization (glutamine) and hydrogen ion buffering (β-alanine), suggesting the universal need to develop these mechanisms in competitive sports. In addition, in both studies, the competition phase strongly differed metabolically from the other phases.

### 4.1. Overtraining Context

Some studies involving endurance athletes suggest that substantial changes in the blood concentrations of specific amino acids may result from and be a sign of overtraining [12,13,14,15,16]. In contrast, other reports did not show adverse changes in amino acid-based indices despite drastic increases in training volume or intensity [28,29], perhaps due to the short periods of training overload that were applied. In this context, none of our athletes were found to be amino acid deficient at any of the four laboratory visits (resting values above the lower limit of the population reference values), and the PFAA concentrations were within the range previously observed in athletes without overtraining symptoms [13]. Moreover, the phenylalanine–tyrosine and glutamine–glutamate ratios were relatively constant throughout the study period and remained outside the range indicative of overtraining [11,13,15]. Pitkänen et al. [10], who, like us, found a training-related decrease in amino acid levels in speed-power athletes, considered such a response to be an expression of a successful adaptive response to training and a normal anabolic state. They reasoned that because the fasting testosterone–cortisol ratio was above the overtraining threshold, physical performance and recovery would not be compromised.

Two studies showed that physically strenuous training periods, even if relatively short (several-week), can result in significant increases in serum sarcosine concentration, accompanied by increases in metabolites such as peroxides, cortisol, and hypoxanthine [30,31]. Accordingly, it was suggested that sarcosine could be one of the markers for the early diagnosis of overreaching/overtraining. In our athletes, resting sarcosine concentrations gradually increased between the transition and competition phases, finally reaching a mean value of 4.75 ± 0.64 μmol∙L^−1^ (3.41–5.40 μmol∙L^−1^ in individual athletes). This was close to the upper limit of medical reference values (~0–5 μmol∙L^−1^), however, essentially still within a normal range. In addition, high resting levels of sarcosine in the competition phase were reduced during progressive exercise more rapidly than in other phases, indicating a more intensive uptake from circulating blood. Sarcosine is an intermediate and byproduct in the synthesis and breakdown of glycine, which occurs naturally in muscle and other body tissues. The metabolic pathways of sarcosine and several other amino acids (glycine, serine, and ethanolamine) are involved in the creatine cycle and the mitochondrial respiratory chain [9,32,33].

Importantly, the levels of other biomarkers were typical of fully fit speed-power athletes. In our participants, the desirable adaptation to the training loads in consecutive phases is evidenced by resting creatine kinase levels characteristic of highly trained athletes [34], stable lactate responses, a decrease in exercise-induced ammonia concentration [35], unaltered hematological indices [36], and sustained maximal oxygen uptake [37]. To this list should be added the successful participation in competitions and satisfactory sprint performance achieved by the athletes at the end of the study period (see Participants section). Previous research shows that such a picture of the above indicators is accompanied by beneficial adaptations involving plasma and red blood cell biomarkers of energy metabolism, adenine nucleotides, hypoxanthine, and the hypoxanthine–guanine phosphoribosyltransferase (HGPRT) enzyme [38,39,40]. Taking all this into account, the response of PFAAs in our study should be considered as a normal physiological adaptation to training load, with no signs of overtraining.

### 4.2. Protein Balance

Two histidine derivatives, 1- and 3-methylhistidine, are the breakdown products of contractile proteins (actin and myosin) [4]. In our study, we observed lower levels and an attenuated response to exercise of these PFAAs during the transition and competition phases, suggesting less exercise-induced protein degradation in response to the same standard exercise test during periods of lower total training load, indicative of a normal adaptive response. This was accompanied by marked increases in skeletal muscle mass (both absolute and percentage) combined with decreases in fat mass throughout the study period. It should be mentioned that during the pre-competition/competition phase, the percentage of energy from carbohydrates, protein, and fat was consistent with nutritional recommendations for athletes, and the protein average intake of 2.2 g·kg^−1^ body weight was within the upper limit of 1.7–2.2 g·kg^−1^ body weight, suggested to be maintained in athletes engaged in intense training [41]. It can therefore be assumed that the nutritional status of the athletes was normal. Although we did not collect analogous data for all training phases and athletes, the partial data obtained at the end of the training cycle suggest the stability of the nutritional status. Taking all this into account, we can assume a positive protein balance and a predominance of synthesis over breakdown in the athletes studied. 

### 4.3. Toxic Ammonia Neutralization

In our study, glutamine levels—pivotal in the ammonia neutralization system—were greatly reduced in the competition phase. At the same time, there were decreases in the levels of citrulline and ornithine, serving as intermediates of the urea cycle, during which highly toxic ammonia is converted to urea and eliminated from the body. This could be seen as a paradox, as it would seem that the levels of glutamine and related amino acids should increase in response to training to cope with the increased workload and neutralize and remove ammonia more efficiently. However, such an interpretation seems overly simplistic and the background of the changes may be different. It was shown that sprint and high-intensity interval training interventions result in attenuated resting adenosine triphosphate (ATP) and total adenine nucleotide levels in skeletal muscle and, importantly, less ATP depletion during exercise [42,43]. Consequently, after training, fewer ‘waste metabolites’ associated with energy production are released in response to exercise, including hypoxanthine and ammonia, with more pronounced adaptations in sprinters than in endurance athletes [35,40]. Therefore, a smaller glutamine amount is needed to neutralize ammonia. It can be assumed that there is an economization of the exercise metabolism and the management of energy resources during sprint-oriented training, for which high-intensity and maximal power exercises are typical. The mechanism of such adaptations may be debatable and is beyond the scope of this study. However, one element is the increased activity of the enzyme HGPRT, which allows for the resynthesis of inosine monophosphate (IMP) from hypoxanthine via a less energy-consuming salvage pathway. In turn, IMP is converted via the purine nucleotide cycle to adenosine monophosphate and further to adenosine diphosphate and ATP [40]. The purine nucleotide cycle involves the amino acid aspartate. Overall, it seems that the reduction in the concentration of specific PFAAs may reflect a beneficial adaptation and optimization of energy mechanisms related to ammonia neutralization and ATP resynthesis.

The profile of blood amino acids (and other metabolites) is an interesting and important biomarker; however, it only indirectly and partially reflects metabolic processes in the human body. In simple terms, the decrease in training-related plasma glutamine concentration we observed, and thus its lower availability in the circulatory system for tissues and organs, may result (i) from a decrease in production and release into the blood or (ii) from an increase in uptake and utilization by tissues and organs. In healthy individuals, there is a balance between the main glutamine producers (skeletal muscle, liver, adipocytes, and lungs) and consumers (immune system, gut, kidneys, and brain), while any kind of metabolic stress, including intense exercise or training, shift this balance toward higher demand for glutamine by the liver, immune system, and gut [44]. Thus, it can be interpreted that in addition to economizing energy metabolism in the skeletal muscle itself (less ammonia production), the analyzed sprint-oriented training caused the redirection of some glutamine resources to the latter recipients to support many other important physiological processes (for example, gluconeogenesis, immune function, or intestinal metabolic activity). It should be emphasized that glutamine is the most prevalent and multifunctional amino acid in the body, being fundamental to many metabolic processes [44]. Furthermore, the decrease in blood glutamine levels with training appears to be independent of the athlete’s condition. One study showed that training caused a significant decrease in resting plasma glutamine concentration in both normally adapted and overtrained athletes, accompanied by an increase in glutamate concentration and a decrease in the glutamine–glutamate ratio in those two groups [15]. This suggests that a training-induced decrease in blood glutamine levels is a typical response, and that considering it normal or abnormal depends on the physiological lower limit criterion adopted and the analysis of other indicators.

### 4.4. Hydrogen Ion Buffering

In this study, we observed decreased plasma levels of histidine and a change in the exercise-induced response of plasma β-alanine in the competition phase. Histidine and β-alanine are precursors of carnosine (β-alanyl-L-histidine) and its derivatives (e.g., anserine), which act as powerful buffers to attenuate the deleterious effects of H+ ions in skeletal muscle cells, produced during high-intensity anaerobic exercise [8]. Much higher levels of carnosine are observed in fast glycolytic (Type IIX) muscle fibers, typical of sprinters, than in oxidative (Type I) muscle fibers, and there is a significant positive correlation between muscle carnosine concentration and power output during maximal sprinting [45,46]. Increased muscle carnosine levels are thought to have an ergogenic effect and reduce fatigue during high-intensity exercise [47]. Importantly, carnosine levels in skeletal muscle increase after sprint training, leading to improved muscle contractility due to better pH buffering and Ca^2+^ sensitivity [48]. It is estimated that more than 99% of the carnosine present in the body is found in skeletal muscle tissue, while in other tissues and body fluids, it is measurable at concentrations 10 to 1000 times lower than in muscle [46] or undetectable, as was the case in our study. We can venture to assume that, as a result of the specialized speed-power training, the highest muscle concentration of carnosine in our sprinters occurred during the competition phase, i.e., during the period of achieving optimal metabolic adaptation and the greatest potential for athletic performance. The lowest plasma histidine levels at this time can be interpreted as its greater extraction from the blood into the muscle due to higher demand and utilization for carnosine synthesis. On the other hand, it is important to remember that histidine is an essential amino acid that is not synthesized in the human body and is only supplied in the diet; therefore, its plasma concentration could also decrease due to less intake with food or dietary supplements. However, this second possibility seems less likely because it was during the competition phase that protein intake was high, and plasma histidine concentration remained at levels typical of highly trained healthy athletes [13]. This suggests that training adaptation was the main stimulus for the decrease in histidine levels.

As for β-alanine, it is the rate-limiting precursor of carnosine, which is synthesized in the liver and available in the extracellular environment via the circulatory system [49,50,51]. In our athletes, during the transition, general, and specific training phases, the plasma β-alanine concentration remained lower, and its response to exercise was weak and flattened. In contrast, during the competition phase, we observed a higher availability (plasma concentration) of β-alanine at rest, followed by a marked decrease during the exercise test and an increase within 15 min after exercise. It seems that during the competition phase, there was an increase in circulating β-alanine stores, which were effectively used during the progressive exercise, i.e., abundantly extracted from the blood into the skeletal muscles. We cannot definitely resolve to what extent this was the effect of diet and supplementation and to what extent this was the effect of training adaptation. The concentration of β-alanine in our athletes significantly exceeded values typical of non-training healthy individuals [52], and it is known that physical activity is associated with increased β-alanine concentrations [53]. Thus, very high plasma β-alanine concentrations, particularly during the competition phase, may reflect the combined effect of intense training stimulating endogenous β-alanine synthesis in the liver and dietary intake.

### 4.5. Potential Practical Applications

Tracking changes in PFAA levels can provide additional support in diagnosing training status and rationalizing nutritional supplementation, taking into account the amino acids most relevant to the type of training. In our sprinters, we observed a specific profile of changes in PFAAs during a long training cycle, clearly different from that of endurance athletes, despite some common attributes [19]. Consequently, unique changes in the PFAA profile should be expected depending on the sports specialty and the associated type of predominant chronic exercise loads. The knowledge of the typical and desirable changes in the PFAA profile over long cycles can be another tool to confirm the correctness of an athlete’s adaptation to the applied training loads. It can also be a support in the diagnosis of possible overtraining. If the concentrations of certain PFAAs or their ratios are outside the ranges typical of the athlete’s physiological state, this can be a warning signal, and supplementation of the most ‘sensitive’ amino acids can be one of the preventive or regenerative interventions. In addition, the subsequent training phases of the annual cycle, characterized by specific training loads, generate different PFAA profiles, with the competition phase appearing to be the most different and critical. This provides an important opportunity to differentiate the assessment of training status and possible supplementation according to the phase of a long-term training cycle, during which athletes are subjected for months to loads consisting of specific configurations of volume, intensity, and recovery.

### 4.6. Limitations and Strengths

Even if the sample size met the minimum criteria for using a two-way repeated-measures ANOVA, a larger number of athletes could probably yield more accurate results. On the other hand, the group studied was homogenous as regards sports specialty, performance level, sex, sports experience, the training cycle being implemented, and other key characteristics; therefore, it can be considered representative of this kind of athlete in general. Also, if statistical significance was achieved, the effect sizes were large; thus, even the small sample size allowed us to clearly show the training effect. Potential biases may relate to the timing of blood sample collection relative to the directly preceding training sessions. In competitive athletes, it is difficult to fully control their training schedules, which are determined by training goals and needs. In practice, only a reduction in loads can be administered (‘light’ training sessions or active recovery). For this reason, the conditions before the subsequent laboratory visits could differ slightly. However, the exercise tolerance and recovery capacity of trained athletes is generally high; thus, reducing loads 1–2 days before the test seems to be sufficient to standardize the physiological status in this cohort.

An interpretive limitation is the lack of complete dietary data, so the possible effect of macronutrient intake on changes in PFAA concentration cannot be fully determined. However, we assume that the main and strongest stimulus was the planned training load, which varied according to the standard rules used in the training periodization of professional athletes. The study included only male athletes. Sex differences in energy substrate utilization, including PFAAs, are to be expected; thus, the results cannot be fully applied to female athletes, who should be included in future studies. In addition, the results and conclusions apply to professional athletes, not to the general population. It is hard to expect similar profound adaptive changes in sedentary or recreationally physically active people who do not apply such strong, prolonged and structured training stimuli. The data do not provide information on muscle production and consumption of PFAAs, which can only be assessed with more invasive techniques that are practically impossible to apply to athletes in training (e.g., muscle damage due to biopsy resulting in exclusion from training or competition).

Strengths include (i) for the first time, a study of the PFAA profile in speed-power athletes examined four times over a long training cycle, (ii) a homogeneous group of athletes in terms of sport specialty and performance level, and (iii) proven and accurate analytical methods.

## 5. Conclusions

The data presented suggest that longer cycles of specialized speed-power training lead to specific changes in the PFAA profile in high-performance athletes. The significant changes include individual PFAAs from both proteinogenic and non-proteinogenic categories. It seems that the observed shifts in PFAA concentrations between the major training phases can be considered a normal physiological long-term adaptation in speed-power sports disciplines. It appears that in sprint-oriented athletes, prolonged periods of specialized training primarily affect those PFAAs that support skeletal muscle metabolic pathways responsible for neutralizing toxic ammonia (glutamine, citrulline, ornithine), attenuating the deleterious effects of H+ ions (histidine, β-alanine) and reducing exercise-induced protein breakdown (1- and 3-methylhistidine) during high-intensity exercise. Thus, the change in the PFAA profile between the detraining and competition phases in sprinters can be viewed as a possible expression of the economization and refinement of pathways associated with the removal of or reduction in excess harmful metabolites formed during exercise. In addition, the picture of changes we obtained can serve as a comparison for further studies on the amino acid response to training stimuli in other athletic cohorts, and it also adds to the knowledge of human adaptations to exercise training.

## Figures and Tables

**Table 1:** Descriptive characteristics and differences between the four consecutive training phases.

	Transition	General	Specific	Competition	ANOVA *p*-Value	η^2^
Weight (kg)	82.0 ± 5.8	82.3 ± 6.5	82.9 ± 6.1	83.4 ± 7.1	0.055	0.24
BMI (kg·m^−2^)	23.9 ± 1.0	23.9 ± 1.3	24.1 ± 1.2	24.3 ± 1.2	0.061	0.23
FM (kg)	10.3 ± 2.2	9.0 ± 1.7 ^#^	8.9 ± 1.8 ^#^	8.8 ± 1.5 ^#^	0.002 *	0.43
FM (%)	12.6 ± 3.1	11.0 ± 2.5 ^#^	10.8 ± 2.5 ^#^	10.7 ± 2.3 ^#^	0.001 *	0.45
LBM (kg)	68.1 ± 6.4	69.7 ± 6.9	70.3 ± 6.5 ^#^	70.9 ± 7.5 ^#^	<0.001 *	0.48
LBM (%)	82.9 ± 3.1	84.6 ± 2.6 ^#^	84.7 ± 2.6 ^#^	84.8 ± 2.3 ^#^	0.001 *	0.46
ALST (kg)	33.0 ± 3.7	34.0 ± 3.9	34.2 ± 3.7	35.0 ± 4.6 ^#^	0.009 *	0.35
RSMI (kg·m^−2^)	9.6 ± 0.8	9.9 ± 0.8	9.9 ± 0.9	10.2 ± 0.9 ^#^	0.006 *	0.37
SMM (kg)	38.4 ± 4.1	39.5 ± 4.5	39.7 ± 4.2	40.7 ± 5.2 ^#^	0.009 *	0.34
SMM (%)	46.7 ± 2.3	47.9 ± 1.9	47.9 ± 2.0	48.6 ± 2.4 ^#^	0.015 *	0.32
WBC (10^9^·L^−1^)	5.31 ± 1.18	4.67 ± 0.79	5.03 ± 1.15	5.33 ± 1.40	0.187	0.16
RBC (10^12^·L^−1^)	4.98 ± 0.43	4.81 ± 0.31	4.76 ± 0.25	4.83 ± 0.36	0.067	0.23
Hb (g∙dL^−1^)	8.84 ± 0.67	8.71 ± 0.40	8.79 ± 0.35	8.91 ± 0.42	0.500	0.08
HCT (%)	42.0 ± 3.1	42.4 ± 2.1	41.5 ± 1.9	42.1 ± 2.4	0.725	0.05
CK (U·L^−1^)	370 ± 328	499 ± 307	401 ± 224	319 ± 122	0.207	0.15
VO_2_max (L·min^−1^)	4.24 ± 0.35	4.37 ± 0.42	4.28 ± 0.43	4.29 ± 0.40	0.715	0.05
VO_2_max (L·min^−1^·kg weight ^−1^)	51.9 ± 4.6	53.4 ± 6.9	51.7 ± 4.4	51.5 ± 3.5	0.513	0.08
VO_2_max (L·min^−1^·kg SMM ^−1^)	111.2 ± 12.2	111.8 ± 18.2	108.3 ± 11.6	106.2 ± 9.7	0.309	0.12
HRmax (bpm)	188 ± 10	190 ± 9	190 ± 7	189 ± 7	0.563	0.07

Abbreviations: BMI—body mass index; ALST—appendicular lean soft tissue; RSMI—relative skeletal muscle index; SMM—skeletal muscle mass; FM—fat mass; LBM—lean body mass; WBC—white blood cells; RBC—red blood cells; Hb—hemoglobin; HCT—hematocrit; CK—creatine kinase; VO_2_max—maximum oxygen uptake; HRmax—maximum heart rate; * significant ANOVA effect; ^#^ different from the transition phase.

**Table 2:** Estimated daily macronutrient intake from diet and dietary supplements during the pre-competition training phase for six of the ten athletes studied.

	Protein	Fat	Carbohydrate
Intake			
g·day^−1^	187 ± 29	161 ± 41	564 ± 139
g·day^−1^·kg^−1^	2.2 ± 0.2	1.9 ± 0.5	6.7 ± 1.2
Energy			
kcal·day^−1^	750 ± 116	1447 ± 366	2178 ± 535
kcal·day^−1^·kg^−1^	8.9 ± 1.0	17.3 ± 4.5	25.8 ± 4.7
% energy intake	17.5 ± 3.0	32.9 ± 5.5	49.6 ± 5.6

**Figure A1:**
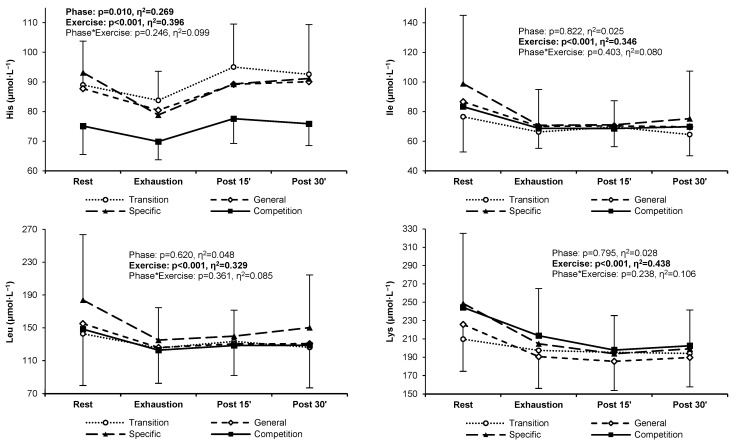

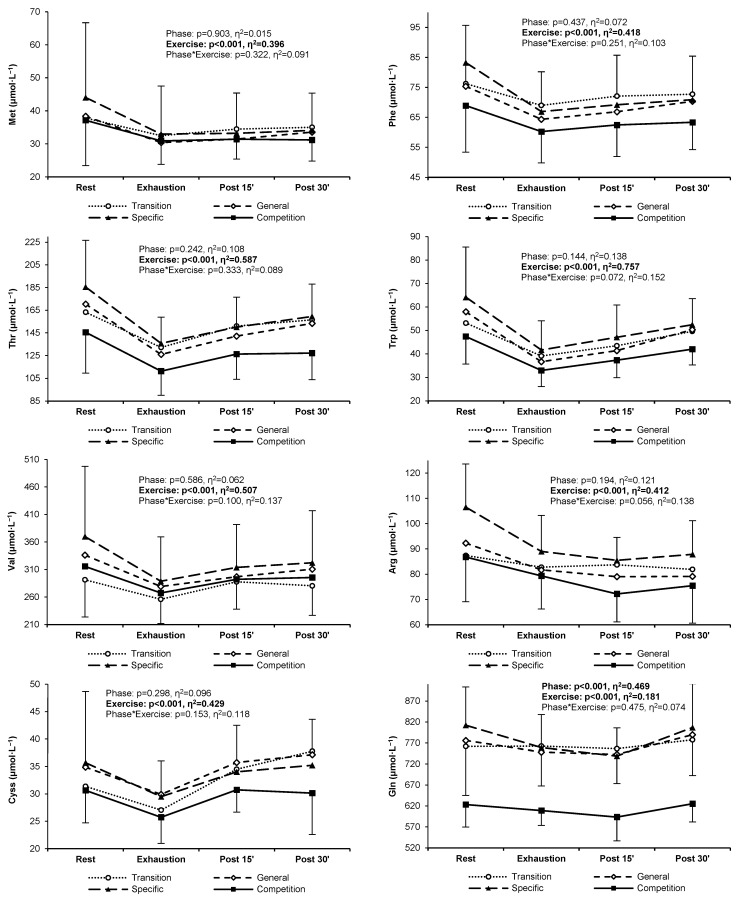

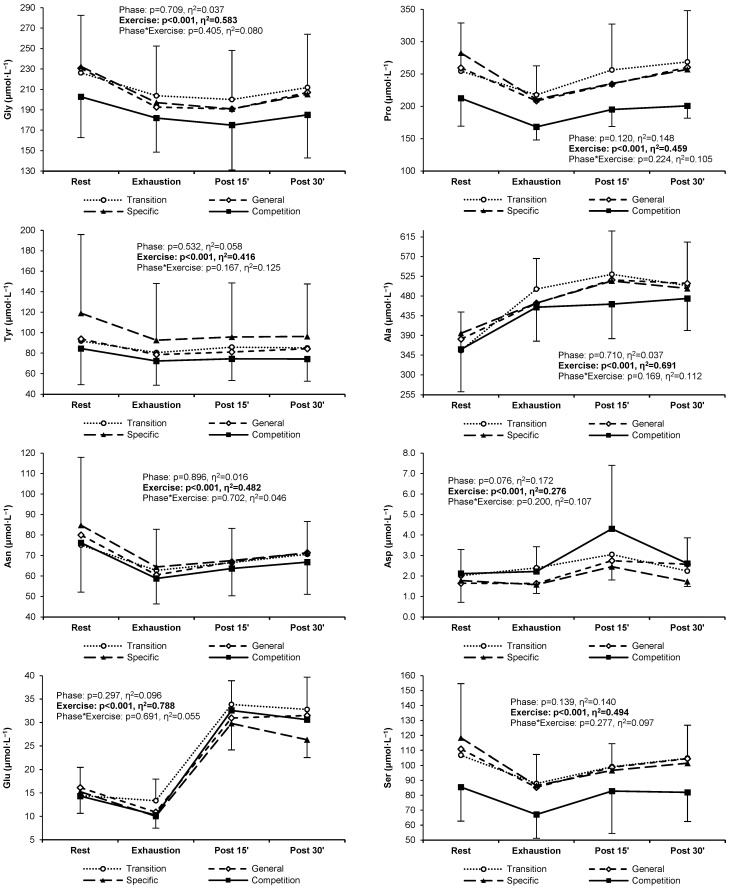

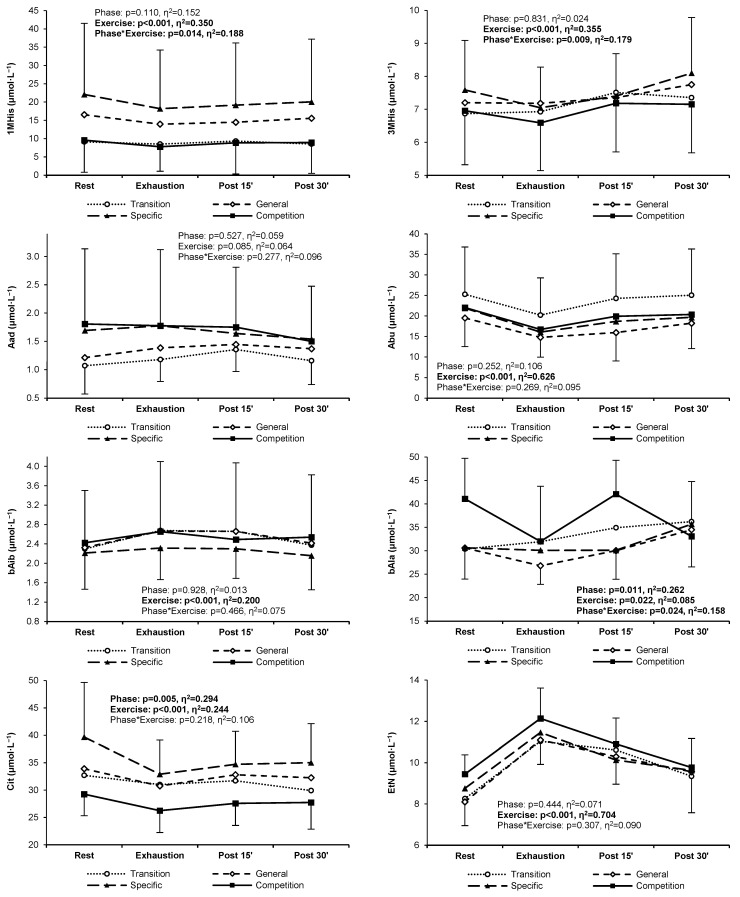

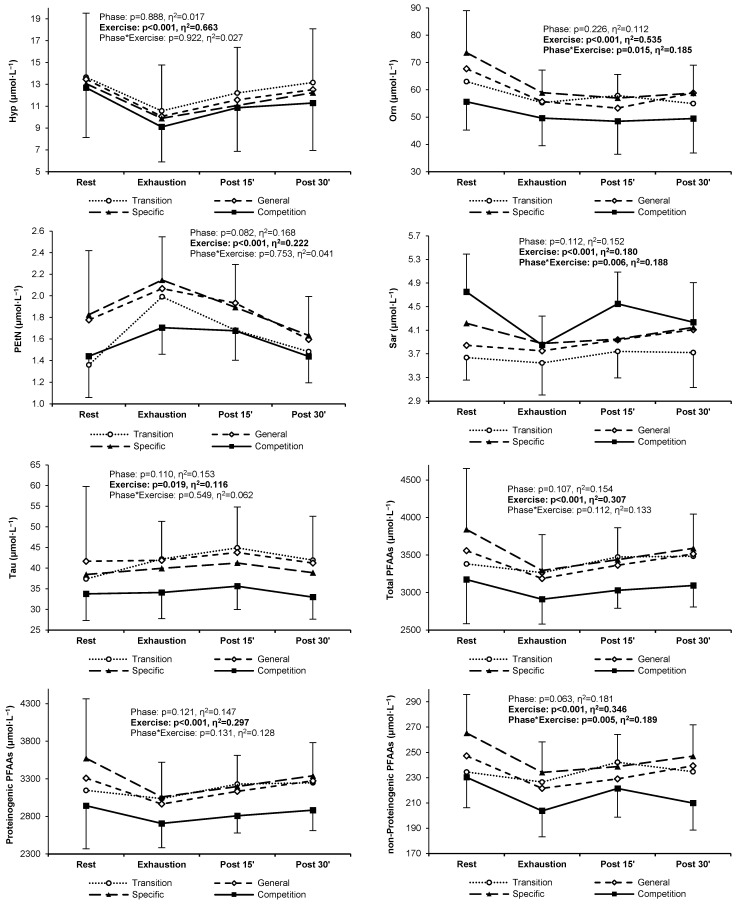

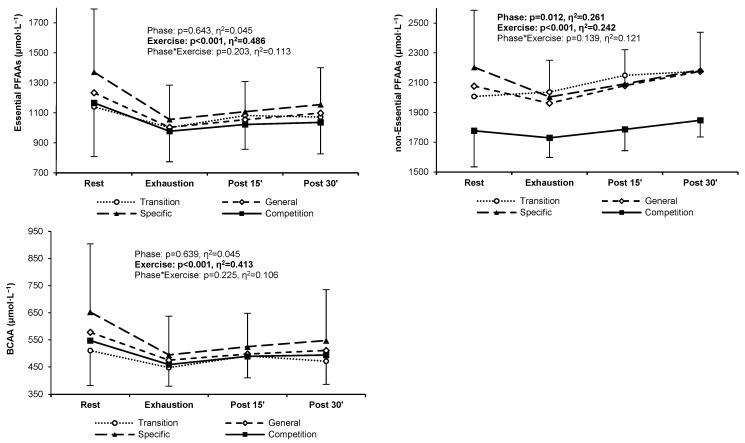
Plasma concentrations of free amino acids in highly trained sprinters at rest, at exhaustion, and during post-exercise recovery after each of the four training phases in a 6-month cycle.

## Data Availability

The original raw data analyzed in this study are included in the Supplementary Materials; further inquiries can be directed to the corresponding author.

## Supplementary Materials

The following supporting information can be downloaded at: https://www.mdpi.com/article/10.3390/jcm13175300/s1, Supplementary File S1: Original raw data.

## Appendix A

**Table A1:** Multiple reaction monitoring (MRM) transitions for 42 amino acids and their corresponding internal standards with applied collision energy values and limits of quantification were achieved by the LC-ESI-MS/MS methodology we used.

No	Amino Acid	Abbrev.	MRM Transition (Q1 → Q3)		Collision Energy (eV)	Limit of Quantitation (μmol∙L^−1^)
			Analyte	Internal Std.		
1	1-methylhistidine	1MHis	318.2 → 121.1	310.2 → 113.1	30	0.2
2	3-methylhistidine	3MHis	318.2 → 121.1	310.2 → 113.1	30	0.2
3	alanine	Ala	238.2 → 121.1	230.2 → 113.1	30	0.2
4	anserine	Ans	389.2 → 121.1	381.2 → 113.1	30	0.5
5	arginine	Arg	323.2 → 121.1	315.2 → 113.1	30	0.5
6	argininosuccinic acid	Asa	439.2 → 121.1	431.2 → 113.1	50	1.0
7	asparagine	Asn	281.2 → 121.1	273.2 → 113.1	30	0.5
8	aspartic acid	Asp	282.1 → 121.1	274.1 → 113.1	30	0.1
9	carnosine	Car	375.2 → 121.1	367.2 → 113.1	30	0.5
10	citrulline	Cit	324.2 → 121.1	316.2 → 113.1	30	0.5
11	cystathionine	Cth	519.3 → 121.1	503.3 → 113.1	50	0.5
12	cystine	Cys	537.2 → 121.1	521.2 → 113.1	50	1.0
13	ethanolamine	EtN	210.2 → 121.1	202.2 → 113.1	30	0.5
14	glutamic acid	Glu	296.2 → 121.1	288.2 → 113.1	30	0.5
15	glutamine	Gln	295.2 → 121.1	287.2 → 113.1	30	0.5
16	glycine	Gly	224.1 → 121.1	216.1 → 113.1	30	1.0
17	histidine	His	304.2 → 121.1	296.2 → 113.1	30	0.5
18	homocitrulline	Hcit	338.2 → 121.1	330.2 → 113.1	30	0.2
19	homocystine	Hcy	565.3 → 121.1	549.3 → 113.1	50	0.5
20	hydroxyproline	Hyp	280.1 → 121.1	272.1 → 113.1	30	0.2
21	isoleucine	Ile	280.2 → 121.1	272.2 → 113.1	30	0.5
22	leucine	Leu	280.2 → 121.1	272.2 → 113.1	30	0.5
23	lysine	Lys	443.3 → 121.1	427.3 → 113.1	50	0.5
24	methionine	Met	298.2 → 121.1	290.2 → 113.1	30	0.1
25	ornithine	Orn	429.3 → 121.1	413.3 → 113.1	50	0.5
26	phenylalanine	Phe	314.2 → 121.1	306.2 → 113.1	30	0.2
27	phosphoethanolamine	PEtN	290.1 → 121.1	282.1 → 113.1	30	0.5
28	phosphoserine	PSer	334.1 → 121.1	326.1 → 113.1	30	0.5
29	proline	Pro	264.2 → 121.1	256.2 → 113.1	30	0.1
30	sarcosine	Sar	238.2 → 121.1	230.2 → 113.1	30	0.2
31	serine	Ser	254.2 → 121.1	246.2 → 113.1	30	0.5
32	taurine	Tau	274.1 → 121.1	266.1 → 113.1	30	0.5
33	threonine	Thr	268.2 → 121.1	260.2 → 113.1	30	0.2
34	tryptophan	Trp	353.2 → 121.1	345.2 → 113.1	30	0.1
35	tyrosine	Tyr	330.2 → 121.1	322.2 → 113.1	30	0.5
36	valine	Val	266.2 → 121.1	258.2 → 113.1	30	0.2
37	α-aminoadipic acid	Aad	310.2 → 121.1	302.2 → 113.1	30	0.2
38	α-amino-n-butyric acid	Abu	252.2 → 121.1	244.2 → 113.1	30	0.2
39	β-alanine	bAla	238.2 → 121.1	230.2 → 113.1	30	0.5
40	β-aminoisobutyric acid	bAib	252.2 → 121.1	244.2 → 113.1	30	0.2
41	γ-amino-n-butyric acid	GABA	252.2 → 121.1	244.2 → 113.1	30	0.05
42	δ-hydroxylysine	Hyl	459.3 → 121.1	443.3 → 113.1	50	0.5

## Appendix C

**Table A2:** Mean values and standard deviations of plasma free amino acids (PFAAs), lactate, and ammonia concentrations in sprint-trained athletes at rest, at exhaustion, and during post-exercise recovery after each of the four training phases in a 6-month cycle.

	Training Phase	Rest		Exhaustion		Recovery			
						15 min		30 min	
		Mean	SD	Mean	SD	Mean	SD	Mean	SD
His	*Transition*	89.0	14.8	83.8	9.8	95.0	14.5	92.6	16.8
	*General*	87.7	11.5	80.5	10.7	89.2	10.9	90.1	15.0
	*Specific*	93.2	11.0	78.9	7.1	89.3	10.6	91.2	15.9
	*Competition*	75.1	9.6	69.9	6.2	77.6	8.4	75.9	7.3
Ile	*Transition*	76.5	23.7	66.3	11.1	69.6	13.2	64.6	14.3
	*General*	86.7	26.7	70.1	17.5	70.3	14.8	69.7	18.3
	*Specific*	98.9	46.2	70.8	24.2	71.0	16.3	75.2	32.1
	*Competition*	83.4	42.7	68.8	23.7	68.6	20.1	69.8	29.8
Leu	*Transition*	143	39	126	15	133	19	126	21
	*General*	155	41	126	25	130	25	131	30
	*Specific*	184	80	135	40	140	32	150	64
	*Competition*	148	68	123	40	128	36	129	52
Lys	*Transition*	210	62	197	46	195	48	194	50
	*General*	226	51	191	34	186	32	190	32
	*Specific*	249	73	205	43	194	41	199	42
	*Competition*	244	81	214	51	198	38	202	39
Met	*Transition*	37.8	15.8	32.5	9.6	34.5	10.5	35.0	10.8
	*General*	38.4	12.5	30.4	7.2	31.4	7.0	33.5	8.8
	*Specific*	44.0	22.7	33.0	14.5	33.2	12.2	34.1	11.3
	*Competition*	37.1	13.7	30.9	7.1	31.4	6.0	31.2	6.4
Phe	*Transition*	76.2	19.5	69.0	11.2	72.1	13.6	72.7	12.7
	*General*	75.4	14.6	64.4	7.9	66.9	8.5	70.4	11.6
	*Specific*	83.3	25.1	66.9	17.9	69.2	15.4	70.9	16.2
	*Competition*	68.9	15.6	60.3	10.4	62.5	10.5	63.3	9.1
Thr	*Transition*	163	54	132	33	151	46	156	52
	*General*	170	54	126	27	142	30	153	32
	*Specific*	185	41	136	23	150	26	159	29
	*Competition*	146	36	111	21	126	22	127	23
Trp	*Transition*	53.1	17.4	39.1	9.2	43.4	10.6	49.6	11.6
	*General*	57.9	10.9	36.6	6.1	41.3	6.5	50.3	6.2
	*Specific*	64.1	21.5	41.7	12.4	47.0	13.8	52.4	11.2
	*Competition*	47.4	11.7	32.9	6.8	37.3	7.4	42.0	6.7
Val	*Transition*	291	68	256	44	288	68	280	54
	*General*	336	89	279	62	297	89	311	72
	*Specific*	370	128	289	81	314	128	322	95
	*Competition*	316	101	267	68	292	101	295	75
Arg	*Transition*	87.4	19.1	82.8	17.0	83.7	15.9	81.9	16.1
	*General*	92.3	21.2	81.7	15.4	79.0	14.8	79.1	14.1
	*Specific*	106.5	17.1	89.0	14.3	85.5	9.1	87.8	13.3
	*Competition*	86.8	17.7	79.3	13.1	72.2	11.1	75.5	14.8
Cyss	*Transition*	31.4	6.0	27.1	6.5	34.5	5.7	37.8	7.5
	*General*	34.9	13.8	29.9	6.1	35.7	6.8	37.2	6.5
	*Specific*	35.7	7.1	29.5	6.3	34.0	7.2	35.2	6.2
	*Competition*	30.6	5.9	25.8	4.8	30.8	4.1	30.2	7.5
Gln	*Transition*	762	117	763	95	756	83	778	85
	*General*	776	104	748	89	743	109	790	99
	*Specific*	812	91	759	79	739	67	807	104
	*Competition*	623	53	609	36	594	57	625	43
Gly	*Transition*	226	56	204	49	200	48	212	52
	*General*	231	72	193	49	191	58	207	64
	*Specific*	233	53	197	44	191	45	205	58
	*Competition*	203	40	182	33	175	44	185	42
Pro	*Transition*	254	75	218	45	256	71	269	79
	*General*	259	83	208	53	234	53	260	64
	*Specific*	283	109	211	56	235	54	257	56
	*Competition*	212	43	168	20	195	26	201	19
Tyr	*Transition*	91.9	40.5	80.5	26.0	85.8	31.5	85.1	29.3
	*General*	93.8	29.6	78.6	20.5	81.1	19.1	84.3	20.5
	*Specific*	119.0	76.9	92.7	55.4	95.7	52.9	96.2	51.4
	*Competition*	84.5	35.1	72.3	23.4	74.4	20.9	74.2	21.4
Ala	*Transition*	356	88	496	70	530	99	504	99
	*General*	381	91	464	55	517	64	509	66
	*Specific*	395	95	464	67	514	90	497	95
	*Competition*	358	96	454	77	461	79	474	73
Asn	*Transition*	75.1	21.6	62.7	13.3	66.5	12.7	70.6	17.0
	*General*	80.0	19.8	60.5	10.0	66.9	10.6	71.3	16.1
	*Specific*	84.8	33.2	64.4	18.3	67.5	15.8	71.3	15.3
	*Competition*	76.2	24.1	58.8	12.5	63.7	13.3	66.7	15.7
Asp	*Transition*	2.02	0.84	2.40	1.03	3.05	0.94	2.24	0.80
	*General*	1.65	0.50	1.63	0.53	2.75	0.63	2.57	1.29
	*Specific*	1.77	1.06	1.58	0.42	2.46	0.65	1.73	0.24
	*Competition*	2.12	1.18	2.22	1.21	4.30	3.10	2.61	1.26
Glu	*Transition*	14.5	6.0	13.3	4.6	33.9	5.1	32.8	6.9
	*General*	16.1	6.0	10.9	4.1	31.0	7.5	31.5	10.2
	*Specific*	15.3	4.6	10.0	2.6	29.8	5.6	26.3	3.7
	*Competition*	14.3	5.8	10.3	3.3	32.6	7.5	30.6	10.3
Ser	*Transition*	106.8	34.2	87.9	23.0	99.1	23.7	104.8	29.8
	*General*	110.9	28.9	85.3	20.4	98.8	22.8	104.5	23.6
	*Specific*	118.4	36.3	86.5	20.7	96.7	17.9	101.5	25.4
	*Competition*	85.5	22.8	67.1	15.9	82.8	28.3	81.9	19.5
1MHis	*Transition*	9.1	8.6	8.5	8.7	9.3	8.5	8.5	8.7
	*General*	16.5	10.5	13.9	9.1	14.5	9.2	15.6	9.9
	*Specific*	22.1	19.4	18.2	16.0	19.1	17.0	20.1	17.1
	*Competition*	9.6	8.8	7.8	6.7	8.8	8.5	8.9	8.4
3MHis	*Transition*	6.37	1.21	6.43	1.39	7.01	1.47	6.86	1.72
	*General*	6.70	1.26	6.68	1.51	6.85	1.39	7.25	1.51
	*Specific*	7.09	1.50	6.55	1.23	6.90	1.29	7.60	1.69
	*Competition*	6.46	1.64	6.09	1.45	6.68	1.48	6.65	1.47
Aad	*Transition*	1.07	0.50	1.18	0.39	1.36	0.39	1.16	0.42
	*General*	1.21	0.56	1.39	0.66	1.45	0.62	1.37	0.56
	*Specific*	1.69	1.21	1.78	1.65	1.64	1.13	1.54	0.92
	*Competition*	1.81	1.33	1.78	1.35	1.75	1.06	1.50	0.97
Abu	*Transition*	25.3	11.5	20.2	9.1	24.3	10.9	25.0	11.3
	*General*	19.5	7.0	14.8	4.8	15.9	6.9	18.2	6.2
	*Specific*	21.9	7.5	16.1	5.1	18.7	6.0	19.7	5.2
	*Competition*	22.0	7.4	16.7	4.8	19.9	5.5	20.3	5.5
bAib	*Transition*	2.30	1.28	2.68	1.48	2.66	1.45	2.37	1.28
	*General*	2.33	1.18	2.67	1.43	2.66	1.42	2.42	1.18
	*Specific*	2.21	0.75	2.32	0.65	2.30	0.61	2.16	0.75
	*Competition*	2.42	0.78	2.65	0.94	2.49	0.97	2.54	0.78
bAla	*Transition*	30.4	7.3	31.9	7.3	34.9	5.5	36.2	9.6
	*General*	30.6	6.6	26.8	4.0	30.0	6.1	34.5	7.9
	*Specific*	30.6	6.4	30.1	6.7	30.1	5.3	35.7	4.8
	*Competition*	41.1	8.6	32.0	11.8	42.1	7.2	33.1	11.7
Cit	*Transition*	32.7	3.3	31.0	3.3	31.7	4.1	29.9	4.1
	*General*	33.9	4.9	30.8	4.4	32.8	4.8	32.3	3.7
	*Specific*	39.7	10.0	32.9	6.2	34.7	6.0	35.0	7.1
	*Competition*	29.2	3.9	26.2	4.0	27.6	4.0	27.7	4.9
EtN	*Transition*	8.26	1.20	11.04	1.51	10.62	1.21	9.35	1.45
	*General*	8.10	1.15	11.10	1.18	10.30	1.33	9.56	1.99
	*Specific*	8.76	1.44	11.46	1.95	10.13	1.44	9.63	1.43
	*Competition*	9.45	0.93	12.14	1.48	10.90	1.26	9.77	1.40
Hyp	*Transition*	13.7	5.9	10.6	4.2	12.2	4.2	13.2	4.9
	*General*	13.5	5.3	10.1	3.9	11.6	4.5	12.5	4.0
	*Specific*	13.1	3.3	9.9	2.8	11.1	3.0	12.2	3.6
	*Competition*	12.7	4.6	9.1	3.2	10.9	4.0	11.3	4.3
Orn	*Transition*	63.0	18.7	55.3	13.9	57.9	14.8	55.0	15.6
	*General*	67.7	15.5	55.7	11.8	53.3	9.6	58.9	14.1
	*Specific*	73.6	15.4	59.0	8.3	57.0	8.6	58.8	10.3
	*Competition*	55.6	10.3	49.6	10.1	48.5	12.1	49.4	12.6
PEtN	*Transition*	1.36	0.39	1.99	0.47	1.68	0.35	1.48	0.34
	*General*	1.78	1.11	2.07	0.41	1.93	0.36	1.59	0.35
	*Specific*	1.83	0.59	2.15	0.40	1.89	0.40	1.63	0.36
	*Competition*	1.44	0.38	1.70	0.25	1.68	0.27	1.44	0.24
Sar	*Transition*	3.64	0.38	3.54	0.54	3.74	0.45	3.72	0.59
	*General*	3.84	0.97	3.75	0.65	3.94	0.78	4.11	0.61
	*Specific*	4.22	1.20	3.88	0.77	3.95	0.63	4.15	0.69
	*Competition*	4.75	0.64	3.85	0.49	4.55	0.54	4.23	0.67
Tau	*Transition*	37.4	5.8	42.2	6.5	44.9	5.4	41.9	7.1
	*General*	41.7	18.1	41.9	9.4	43.8	11.0	41.2	11.3
	*Specific*	38.5	8.1	39.9	7.8	41.2	8.2	38.9	8.2
	*Competition*	33.8	6.5	34.1	6.3	35.6	5.7	33.0	5.4
Proteinogenic	*Transition*	3147	655	3038	374	3231	471	3249	517
	*General*	3311	573	2966	304	3134	337	3275	378
	*Specific*	3575	791	3060	462	3199	415	3341	441
	*Competition*	2943	574	2707	325	2808	229	2883	273
Essential	*Transition*	1140	292	1002	157	1082	196	1072	202
	*General*	1234	265	1004	146	1054	145	1098	175
	*Specific*	1371	421	1055	229	1107	202	1155	246
	*Competition*	1166	357	977	203	1022	165	1036	209
non-Essential	*Transition*	2007	377	2037	235	2149	297	2177	328
	*General*	2077	358	1962	197	2080	239	2177	247
	*Specific*	2204	382	2004	246	2091	230	2187	251
	*Competition*	1777	242	1730	131	1786	142	1847	111
BCAA	*Transition*	511	128	448	68	491	80	471	85
	*General*	578	153	475	101	498	102	511	117
	*Specific*	653	251	495	143	525	124	548	188
	*Competition*	547	210	459	129	489	112	494	151
non-Proteinogenic	*Transition*	235	23	227	25	242	28	235	37
	*General*	247	39	222	24	229	26	239	20
	*Specific*	265	31	234	24	239	25	247	25
	*Competition*	230	24	204	21	221	23	210	21
Total PFAAs	*Transition*	3382	672	3265	389	3473	490	3484	543
	*General*	3558	601	3187	323	3363	352	3514	393
	*Specific*	3841	813	3294	478	3437	427	3588	457
	*Competition*	3173	588	2911	332	3029	240	3093	285
Lactate	*Transition*	1.34	0.50	11.07	1.21	7.12	1.93	4.50	1.55
	*General*	1.34	0.57	10.42	1.67	6.32	1.69	3.66	1.51
	*Specific*	1.11	0.33	10.21	1.25	6.14	1.98	3.30	1.41
	*Competition*	0.94	0.19	9.75	1.30	5.22	1.30	2.67	0.92
Ammonia	*Transition*	31.5	4.0	92.8	8.0	62.4	8.5	40.0	5.6
	*General*	28.3	3.7	80.2	7.1	53.0	8.4	35.7	4.6
	*Specific*	25.9	2.3	70.6	7.0	45.5	5.1	32.2	4.9
	*Competition*	22.8	4.8	66.2	4.3	43.1	4.6	27.6	4.4
Phe:Tyr ratio	*Transition*	0.90	0.18	0.91	0.19	0.90	0.19	0.91	0.19
	*General*	0.84	0.15	0.85	0.17	0.85	0.17	0.86	0.17
	*Specific*	0.77	0.16	0.79	0.17	0.80	0.18	0.81	0.20
	*Competition*	0.86	0.15	0.87	0.16	0.87	0.16	0.89	0.16
Gln:Glu ratio	*Transition*	59.1	22.3	63.6	24.5	22.8	4.0	24.5	5.4
	*General*	54.3	23.9	79.1	34.0	25.9	9.9	27.6	9.8
	*Specific*	56.2	12.0	81.0	24.8	25.9	6.4	31.7	9.4
	*Competition*	48.6	15.9	63.9	16.7	19.2	5.0	22.5	7.3

## References

[1] Gibala M.J. (2001). Regulation of skeletal muscle amino acid metabolism during exercise. Int. J. Sport Nutr. Exerc. Metab..

[2] Felig P., Wahren J. (1971). Amino acid metabolism in exercising man. J. Clin. Investig..

[3] Henriksson J. (1991). Effect of exercise on amino acid concentrations in skeletal muscle and plasma. J. Exp. Biol..

[4] Ishikura K., Ra S.-G., Ohmori H. (2013). Exercise-induced changes in amino acid levels in skeletal muscle and plasma. J. Phys. Fit. Sports Med..

[5] Kamei Y., Hatazawa Y., Uchitomi R., Yoshimura R., Miura S. (2020). Regulation of skeletal muscle function by amino acids. Nutrients.

[6] Rennie M.J., Tipton K.D. (2000). Protein and amino acid metabolism during and after exercise and the effects of nutrition. Ann. Rev. Nutr..

[7] Wagenmakers A.J.M., Richter E.A., Kiens B., Galbo H., Saltin B. (1998). Protein and Amino Acid Metabolism in Human Muscle. Skeletal Muscle Metabolism in Exercise and Diabetes. Advances in Experimental Medicine and Biology.

[8] Abe H. (2013). Role of histidine-related compounds as intracellular proton buffering constituents in vertebrate muscle. Biochemistry.

[9] Wyss M., Kaddurah-Daouk R. (2000). Creatine and creatinine metabolism. Physiol. Rev..

[10] Pitkänen H., Mero A., Oja S.S., Komi P.V., Rusko H., Nummela A., Saransaari P., Takala T. (2002). Effects of training on the exercise-induced changes in serum amino acids and hormones. J. Strength Cond. Res..

[11] Askanazi J., Carpentier Y.A., Michelsen C.B., Elwyn D.H., Furst P., Kantrowitz L.R., Gump F.E., Kinney J.M. (1980). Muscle and plasma amino acids following injury. Influence of intercurrent infection. Ann. Surg..

[12] Ikonen J.N., Joro R., Uusitalo A.L., Kyröläinen H., Kovanen V., Atalay M., Tanskanen-Tervo M.M. (2020). Effects of military training on plasma amino acid concentrations and their associations with overreaching. Exp. Biol. Med..

[13] Kingsbury K.J., Kay L., Hjelm M. (1998). Contrasting plasma free amino acid patterns in elite athletes: Association with fatigue and infection. Br. J. Sports Med..

[14] Krause S., Langrock M., Weiss M. (2002). Influence of seasonal variations in training loads on selected amino acids and parameters of the psychoimmunological network in a swimming team. Int. J. Sports Med..

[15] Smith D.J., Norris S.R. (2000). Changes in glutamine and glutamate concentrations for tracking training tolerance. Med. Sci. Sports Exerc..

[16] Strüder H.K., Hollmann W., Platen P., Wöstmann R., Weicker H., Molderings G.J. (1999). Effect of acute and chronic exercise on plasma amino acids and prolactin concentrations and on [3H]ketanserin binding to serotonin2A receptors on human platelets. Eur. J. Appl. Physiol. Occup. Physiol..

[17] van den Baar M.T., Fekkes D., van den Hoogenband C.R., Duivenvoorden H.J., Pepplinkhuizen L. (2004). Plasma amino acids and sports injuries. Amino Acids.

[18] Gwin J.A., Hatch-McChesney A., Pitts K.P., O’Brien R.P., Karis A.J., Carrigan C.T., McClung J.P., Karl J.P., Margolis L.M. (2022). Initial military training modulates serum fatty acid and amino acid metabolites. Physiol. Rep..

[19] Kusy K., Ciekot-Sołtysiak M., Matysiak J., Klupczyńska-Gabryszak A., Plewa S., Zarębska E.A., Kokot Z.J., Dereziński P., Zieliński J. (2024). Changes in plasma free amino acid profile in endurance athletes over a 9-month training cycle. Metabolites.

[20] Sugimoto T., Kamei Y. (2022). Regulation of skeletal muscle function by amino acids, especially by non-proteinogenic amino acids. J. Nutr. Sci. Vitaminol..

[21] Bompa T., Buzzichelli C.A. (2018). Periodization. Theory and Methodology of Training.

[22] Szponar L., Wolnicka K., Rychlik E. (2000). Album of Photographs of Food Products and Dishes.

[23] Trinschek J., Zieliński J., Zarębska E.A., Kusy K. (2023). Male and female athletes matched for maximum oxygen uptake per skeletal muscle mass: Equal but still different. J. Sports Med. Phys. Fitness.

[24] Kim J., Wang Z., Heymsfield S.B., Baumgartner R.N., Gallagher D. (2002). Total-body skeletal muscle mass: Estimation by a new dual-energy X-ray absorptiometry method. Am. J. Clin. Nutr..

[25] Matysiak J., Dereziński P., Klupczyńska A., Matysiak J., Kaczmarek E., Kokot Z.J. (2014). Effects of a honeybee sting on the serum free amino acid profile in humans. PLoS ONE.

[26] Matomäki P., Kainulainen H., Kyröläinen H. (2018). Corrected whole blood biomarkers—The equation of Dill and Costill revisited. Physiol. Rep..

[27] Faul F., Erdfelder E., Lang A.-G., Buchner A. (2007). G*Power 3: A flexible statistical power analysis program for the social, behavioral, and biomedical sciences. Behav. Res. Methods.

[28] Tanaka H., West K.A., Duncan G.E., Bassett D.R. (1997). Changes in plasma tryptophan/branched chain amino acid ratio in responses to training volume variation. Int. J. Sports Med..

[29] Lehmann M., Mann H., Gastmann U., Keul J., Vetter D., Steinacker J.M., Häussinger D. (1996). Unaccustomed high-mileage vs intensity training-related changes in performance and serum amino acid levels. Int. J. Sports Med..

[30] Felder T.K., Ring-Dimitriou S., Auer S., Soyal S.M., Kedenko L., Rinnerthaler M., Cadamuro J., Haschke-Becher E., Aigner E., Paulweber B. (2017). Specific circulating phospholipids, acylcarnitines, amino acids and biogenic amines are aerobic exercise markers. J. Sci. Med. Sport.

[31] Loureiro L.L., Ferreira T.J., da Costa C.S.C., Fidalgo T.K.S., Valente A.P., Pierucci A.P.T.R. (2022). Impact of precompetitive training on metabolites in modern pentathletes. Int. J. Sports Physiol. Perform..

[32] Glorieux F.H., Scriver C.R., Delvin E., Mohyuddin F. (1971). Transport and metabolism of sarcosine in hypersarcosinemic and normal phenotypes. J. Clin. Investig..

[33] Moolenaar S.H., Poggi-Bach J., Engelke U.F., Corstiaensen J.M., Heerschap A., de Jong J.G., Binzak B.A., Vockley J., Wevers R.A. (1999). Defect in dimethylglycine dehydrogenase, a new inborn error of metabolism: NMR spectroscopy study. Clin. Chem..

[34] Mougios V. (2007). Reference intervals for serum creatine kinase in athletes. Br. J. Sports Med..

[35] Włodarczyk M., Kusy K., Słomińska E.M., Krasiński Z., Zieliński J. (2020). Change in lactate, ammonia, and hypoxanthine concentrations in a 1-year training cycle in highly trained athletes: Applying biomarkers as tools to assess training status. J. Strength Cond. Res..

[36] Ciekot-Sołtysiak M., Kusy K., Podgórski T., Zieliński J. (2018). Training-induced annual changes in red blood cell profile in highly-trained endurance and speed-power athletes. J. Sports Med. Phys. Fitness.

[37] Trinschek J., Zieliński J., Kusy K. (2020). Maximal oxygen uptake adjusted for skeletal muscle mass in competitive speed-power and endurance male athletes: Changes in a one-year training cycle. Int. J. Environ. Res. Public Health.

[38] Pospieszna B., Kusy K., Słomińska E.M., Dudzinska W., Ciekot-Sołtysiak M., Zieliński J. (2020). The effect of training on erythrocyte energy status and plasma purine metabolites in athletes. Metabolites.

[39] Zarębska E.A., Kusy K., Słomińska E.M., Kruszyna Ł., Zieliński J. (2019). Alterations in exercise-induced plasma adenosine triphosphate concentration in highly trained athletes in a one-year training cycle. Metabolites.

[40] Zieliński J., Kusy K. (2012). Training-induced changes in purine metabolism in high-level sprinters vs triathletes. J. Appl. Physiol..

[41] Kerksick C.M., Wilborn C.D., Roberts M.D., Smith-Ryan A., Kleiner S.M., Jäger R., Collins R., Cooke M., Davis J.N., Galvan E. (2018). ISSN exercise & sports nutrition review update: Research & recommendations. J. Int. Soc. Sports Nutr..

[42] Stathis C.G., Febbraio M.A., Carey M.F., Snow R.J. (1994). Influence of sprint training on human skeletal muscle purine nucleotide metabolism. J. Appl. Physiol..

[43] Hellsten-Westing Y., Norman B., Balsom P.D., Sjödin B. (1993). Decreased resting levels of adenine nucleotides in human skeletal muscle after high-intensity training. J. Appl. Physiol..

[44] Cruzat V., Macedo Rogero M., Noel Keane K., Curi R., Newsholme P. (2018). Glutamine: Metabolism and immune function, supplementation and clinical translation. Nutrients.

[45] Boldyrev A.A., Aldini G., Derave W. (2013). Physiology and pathophysiology of carnosine. Physiol. Rev..

[46] Suzuki Y., Ito O., Mukai N., Takahashi H., Takamatsu K. (2002). High level of skeletal muscle carnosine contributes to the latter half of exercise performance during 30-s maximal cycle ergometer sprinting. Jpn. J. Physiol..

[47] Hill C.A., Harris R.C., Kim H.J., Harris B.D., Sale C., Boobis L.H., Kim C.K., Wise J.A. (2007). Influence of beta-alanine supplementation on skeletal muscle carnosine concentrations and high intensity cycling capacity. Amino Acids.

[48] Parkhouse W.S., McKenzie D.C., Hochachka P.W., Ovalle W.K. (1985). Buffering capacity of deproteinized human vastus lateralis muscle. J. Appl. Physiol..

[49] Artioli G.G., Sale C., Jones R.L. (2019). Carnosine in health and disease. Eur. J. Sport Sci..

[50] Brisola G.M.P., Zagatto A.M. (2019). Ergogenic effects of β-alanine supplementation on different sports modalities: Strong evidence or only incipient findings?. J. Strength Cond. Res..

[51] Wu G. (2022). Amino Acids in Nutrition and Health. Amino Acids in Systems Function and Health.

[52] Church D.D., Hoffman J.R., Varanoske A.N., Wang R., Baker K.M., La Monica M.B., Beyer K.S., Dodd S.J., Oliveira L.P., Harris R.C. (2017). Comparison of two β-alanine dosing protocols on muscle carnosine elevations. J. Am. Coll. Nutr..

[53] Zhang Y., Liu D., Ma Z., Wang C., Gu S., Zhou Z., Zuo H. (2023). Plasma β-alanine is positively associated with risk of ischemic stroke: A nested case-control study. J. Nutr..

